# Supplementary material for: Discovery and Characterization of a Dual-Function Peptide Derived from Bitter Gourd Seed Protein Using Two Orthogonal Bioassay-Guided Fractionations Coupled with In Silico Analysis
Source: Pharmaceuticals (Basel). 2023 Nov 20;16(11):1629. doi: 10.3390/ph16111629 (PMC10674851; doi:10.3390/ph16111629)
Supplement: Supplementary file 1 [file pharmaceuticals-16-01629-s001.zip › pharmaceuticals-2676466-supplementary.pdf]

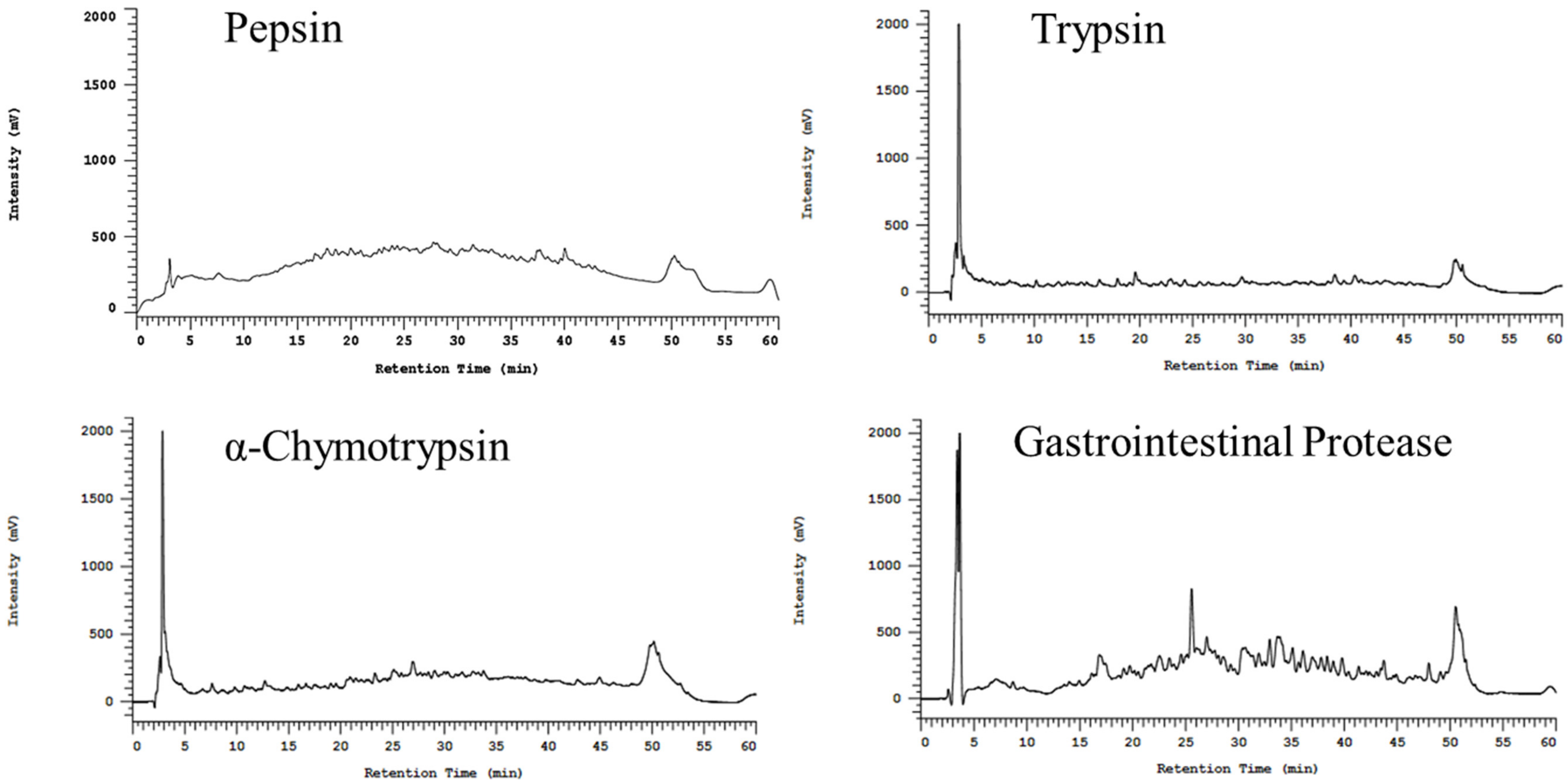

Figure S1. RP-HPLC chromatogram of 50 µg/µL BGSP hydrolysate generated from pepsin, trypsin, α-chymotrypsin, and gastrointestinal protease

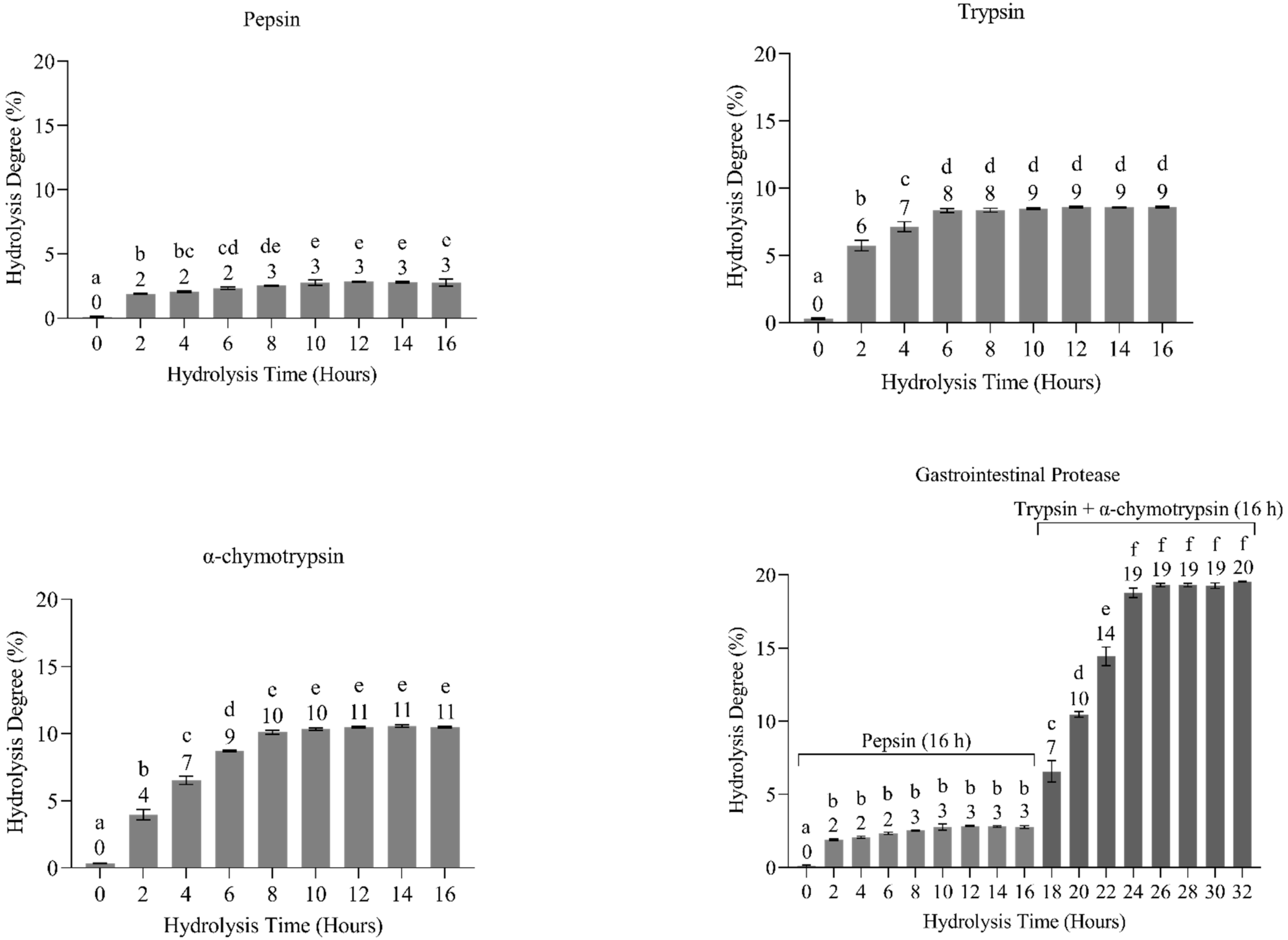

Figure S2. Hydrolysis Degree of BGSP hydrolysate generated from pepsin, trypsin, α-chymotrypsin, and gastrointestinal protease. Different letters mean significantly different ($p<0.05$) of liberated amino acids at certain hydrolysis times compared to the control (0 hours).

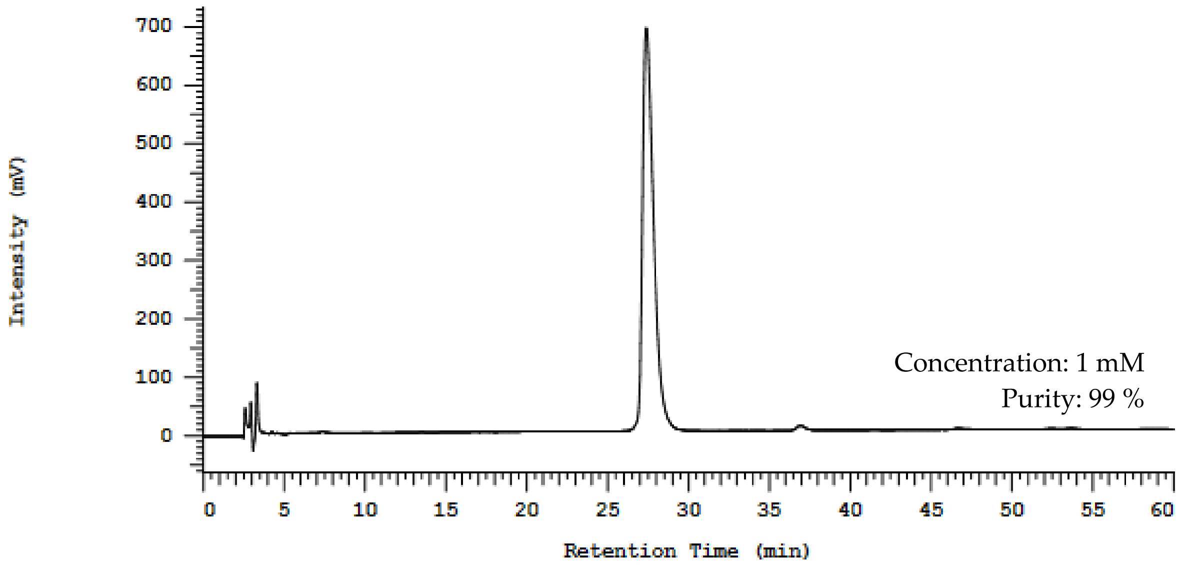

Figure S3. RP-HPLC chromatogram of synthetic AW6

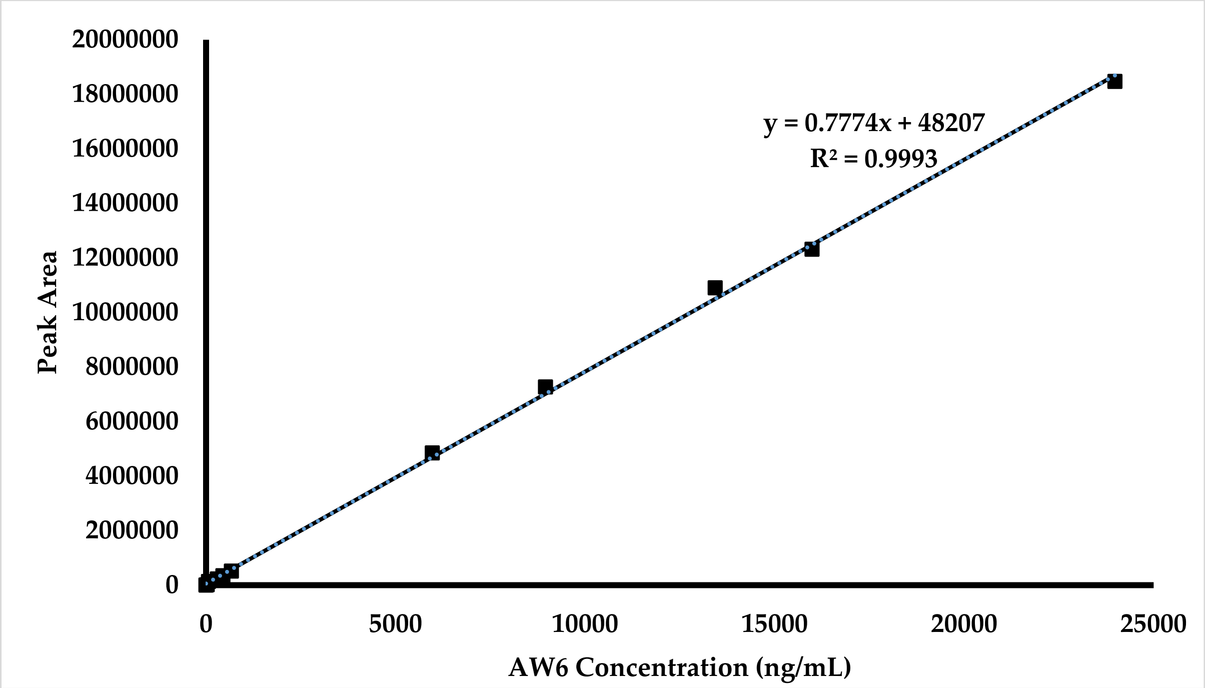

Figure S4. The standard calibration curve of AW6

Table S1. Identified peptides from GP BGSP hydrolysate fraction F5 from RP-HPLC fractionation

Identified Protein	Peptide Sequence	m/z (Observed)	Mass	Peptide Length
(gi|157829880) chain A, alpha-momocharin (sequence coverage 28%)	PALDSAISTL	494.2716	986.5284	10
	DEVPSLATI	944.4933	943.4862	9
	ATISLENSW	1020.5003	1019.4924	9
	TISLENSW	949.4633	948.4553	8
	VVTSNIQLL	493.7979	985.5808	9
	IQLAQGNNGIF	587.8142	1173.6143	11
	EKIPIGL	385.2444	768.4745	7
	IPIGL	512.3445	511.3370	5
	DSAISTLL	819.4460	818.4385	8
	ISLENSW	848.4136	847.4076	7
	ITLPY	606.3495	605.3425	5
	VYNIPL	718.4134	717.4061	6
	AAAGAL	473.2718	472.2645	6
(gi|1229803397) legumin A-like [Momordica charantia] (sequence coverage 18%)	IQVVDDRGQTIF	695.8691	1389.7252	12
	NIIKVEGPL	982.5919	981.5858	9
	YIIEPNGL	918.4926	917.4858	8
	AAEEGLEW	904.4037	903.3974	8
	IIEPNGLL	868.5144	867.5065	8
	ALPVQVI	739.4713	738.4639	7
	PAGATHW	739.3508	738.3449	7
	TLAQIL	658.4133	657.4061	6
	IIKVEGPL	434.7789	867.5429	8
	EGLEW	633.2882	632.2806	5
	HSIIF	308.6762	615.3380	5
(gi|1229809665) superoxide dismutase [Cu-Zn] [Momordica	GASDVVSGTIF	1052.5234	1051.5186	11
	AVAVL	472.2747	471.3057	5
	DVVSGTIF	837.4351	836.4280	8

charantia] (sequence coverage 11%)				
	VVVAGEF	720.3925	719.3854	7
	NVANQLDPYL	573.7944	1145.5718	10
	EPTTSDVVVAGEFDQGSGSMR	1084.9948	2167.9690	21
	TLAQLL	658.4130	657.4061	6
	QLFPR	330.6841	659.3755	5
	LFGSL	536.3083	535.3006	5
	APLVSW	672.3657	671.3643	6
**de novo* sequencing (only)	QGLVSW	689.3613	688.3544	6
	NLDLGL	644.3618	643.3541	6
	LVVEL	572.3657	571.3581	5
	QGKK	230.7098	459.2805	4
	LPLGL	512.3439	511.3370	5
	APLLSDSF	849.4352	848.4279	8
	ALVNAF	634.3193	633.3486	6
	HSAGF	518.2610	517.2285	5
	SPVWVY	375.6943	749.3748	6

**de novo* sequencing (only): PEAKS executed *de novo* peptide sequencing based on LC-MS/MS raw data without being assigned to the protein database matching

Table S2. Identified peptides from GP BGSP hydrolysate fraction S1 from SCX fractionation

Identified Protein	Peptide Sequence	m/z (Observed)	Mass	Peptide Length
(gi|157829880) chain A, alpha-momocharin (sequence coverage 6%)	ATISLENSW	1020.4997	1019.4924	9
	TPIVL	542.3547	541.3475	5
(gi|1229803397) legumin A-like [Momordica charantia] (sequence coverage 4%)	ALPVQVIASAY	566.3237	1130.6335	11
	FAVPAGATHW	528.7667	1055.5188	10
(gi|1229803542) 11S globulin seed storage protein 2-like [Momordica charantia] (sequence coverage 5%)	IVVAF	548.3444	547.3370	5
	IVAVSAGTVEW	1131.6045	1130.5972	11
	VIPQFY	766.4132	765.4061	6
(gi|1229778196) aspartic proteinase-like [Momordica charantia] (sequence coverage 3%)	ILGDIF	677.3859	676.3795	6
*de novo sequencing (only)	VVVAGEF	720.3921	719.3854	7
	WLVPSVY	862.9591	862.4589	7
	NVANQLDPYL	573.7931	1145.5718	10
	WELTL	661.3743	660.3483	5
	LFLPPF	733.4279	732.4210	6
	EPTTSDVVVAGEFDQGSGSMR	1084.9946	2167.9690	21
	VFLPQF	750.4175	749.4112	6
	SLPDLVF	790.9355	789.4272	7
	NLPLLL	682.4500	681.4425	6
	APLVSW	672.3745	671.3663	6

**de novo* sequencing (only): PEAKS executed *de novo* peptide sequencing based on LC-MS/MS raw data without being assigned to the protein database matching

Table S3. The comparison of molecular interaction between lisinopril and AW6 on ACE (PDB code = 1O86)

ACE Catalytic Site		Lisinopril		APLVSW	
Pocket	Amino Acid Residues	Interaction	Distance (Å)	Interaction	Distance (Å)
S1	Ala354	H-Bond	2.9	-	-
	Glu384	H-Bond	2.7	-	-
	Tyr523	H-Bond	2.8	H-Bond (Ser5 side chain)	1.9
S1'	Glu162	H-Bond	3.4	-	-
S2'	Gln281	H-Bond	2.2	-	-
	His353	H-Bond	2.8	H-Bond (Trp6 main chain)	2.3
	Lys511	H-Bond	2.9	Ionic (Trp6 main chain)<br>π-π (Trp6 side chain)	3.3<br>6.8
	His513	H-Bond	3.1	H-Bond (Ser5 side chain)	2.2
	Tyr520	H-Bond	2.6	H-Bond (Trp6 main chain)	2.3
Non-Catalytic Site	Glu143	-	-	H-Bond (Ala1 main chain)<br>H-Bond (Ala1 main chain)<br>Ionic (Ala1 main chain)	2.4<br>2.3<br>2.8
	Asn70	-	-	H-Bond (Ala1 main chain)	2.5

Table S4. The comparison of molecular interaction between diprotin A and AW6 toward DPP4 (PDB code = 1WCY)

DPP4 Catalytic Site		Diprotin A		APLVSW (AW6)	
Pocket	Amino Acid Residues	Interaction	Distance (Å) Molecule B	Interaction	Distance (Å)
S2	Glu205	H-Bond	2.73	-	-
	Glu206	H-Bond	2.65	-	-
	Tyr662	H-Bond	3.00	-	-
S1	Tyr547	H-Bond	2.68	-	-
	Tyr631	H-Bond	2.98	-	-
S1’	Arg125	H-Bond	2.99	-	-
Non-catalytic Site	Ser59	-	-	H-Bond	2.00
	Ile407	-	-	H-Bond	1.80
	Arg471	-	-	H-Bond<br>Charge	1.90<br>2.50
	Glu408	-	-	H-Bond<br>Charge	1.80<br>2.80
